# Spontaneous ventral gallbladder hernia complicated with perforation; a case report and literature review

**DOI:** 10.1016/j.ijscr.2023.108314

**Published:** 2023-05-10

**Authors:** Majid Samsami, Seyed Pedram Kouchak Hoseini, Hojatolah Khoshnoudi, Mohammad Aghaei, Fatemeh Parsaeian, Alireza Haghbin Toutounchi

**Affiliations:** Department of General Surgery, Imam Hosein Medical and Educational Center, Shahid Beheshti University of Medical sciences, Tehran, Iran

**Keywords:** Case report, Ventral hernia, Gallbladder herniation, Cholecystitis, Abdominal wall hernia

## Abstract

**Introduction and importance:**

Ventral gallbladder hernia is a rare condition mostly related to past acquired abdominal wall defects, but spontaneous ones are scarce. It happens more in elderly patients. Etiology and causes are still unspecified, but the most related known causes of spontaneous gallbladder herniation are carcinoma, biliary tracked occlusion or abdominal wall weakness in elderly patients, respectively.

**Case presentation:**

We have presented a complicated 90-year-old woman with a bulged and warm area at the right upper abdomen with tenderness and positive rebound tenderness. In help with imaging, we found a ventral gallbladder hernia perforated in the subcutaneous layer. Then cholecystectomy and herniation site repair was performed.

**Clinical discussion:**

We have explained this infrequent scenario and reviewed recent similar papers to find further relevant information. The common presentations, probable causes, the role of imaging in diagnosis and the management are discussed for the best surgical planning.

**Conclusion:**

The spontaneous ventral herniation of the gallbladder is an exceedingly uncommon occurrence. The diagnosis of this condition heavily relies on imaging, with computed tomography (CT) scan utilizing both intravenous and oral contrast being the optimal modality. Management of this condition can be accomplished via both laparoscopic and laparotomy approaches. It is our recommendation to perform cholecystectomy and hernia repair simultaneously and expeditiously in all cases. We advise against conservative management strategies.

## Highlights

• Spontaneous ventral gallbladder herniation is extremely rare.
• Imaging has an essential role in diagnosis and the best modality is a CT scan with IV and oral contrast.
• Overall, it occurs more in elderly female patients.
• We believe that the ventral gallbladder hernia must be repaired at the same time when the cholecystectomy is performed.
• In management, the most crucial point is to avoid reducing the hernia if gallbladder herniation is suspected.

## 1 Introduction

Abdominal hernia is a protrusion of peritoneal contents through the abdominal wall [1]. Ventral gallbladder hernia is a rare kind of abdominal wall hernia. In literature, just a few cases are described with an incisional hernia, intraperitoneal hernia, like protrusion into the foramen of Winslow, Parastomal, or even an inguinal protrusion. However, based on our search, the spontaneous gallbladder ventral hernia with no past defect remains extremely rare. Overall, it occurs more in elderly female patients [1]. We have presented a perforated spontaneous gallbladder hernia which was incarcerated through the abdominal wall. This article has been reported in line with the SCARE 2020 criteria [11].

## 2 Presentation of case

### 2.1 History

A 90-year-old woman presented to the emergency department with latent nausea, vomiting and abdominal pain. She was ill in general appearance. She had diabetes and hypertension in her past medical history and no past surgery. Her chronic diseases were poorly controlled.

### 2.2 Assessment

**Fig. 1:**
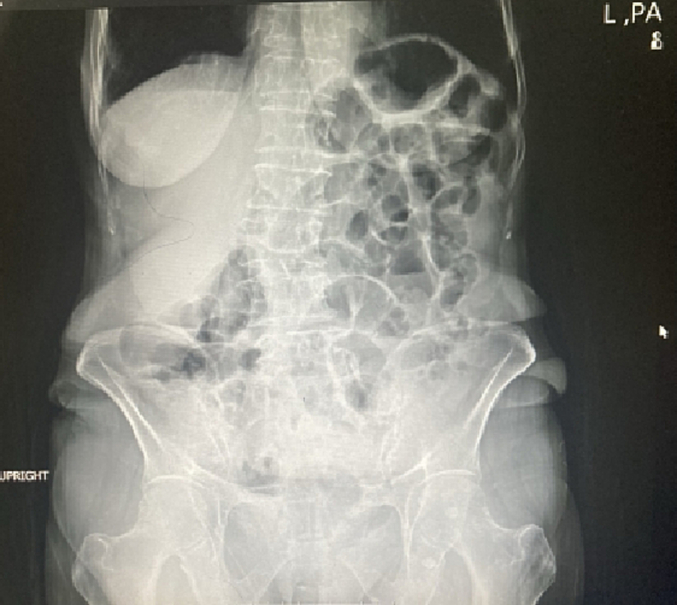
The herniated gallbladder in up right.

**Fig. 2:**
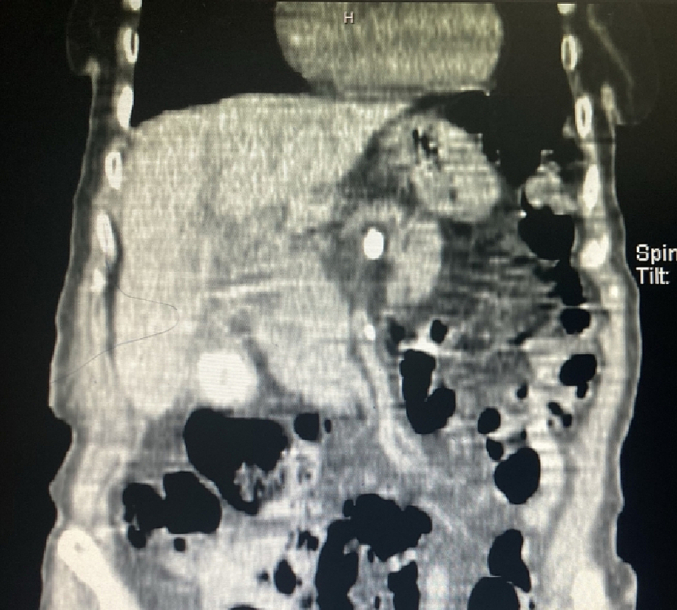
The gallstone in fondus.

**Fig. 3:**
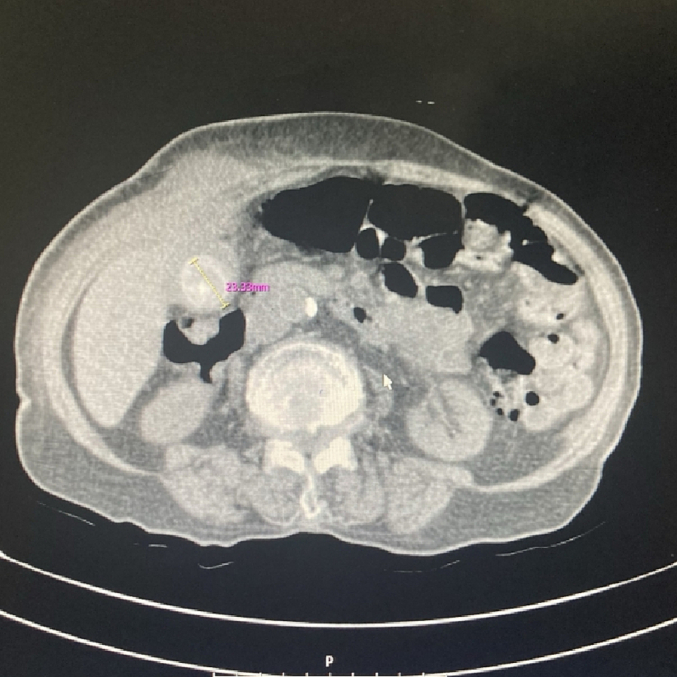
The stone.

**Fig. 4:**
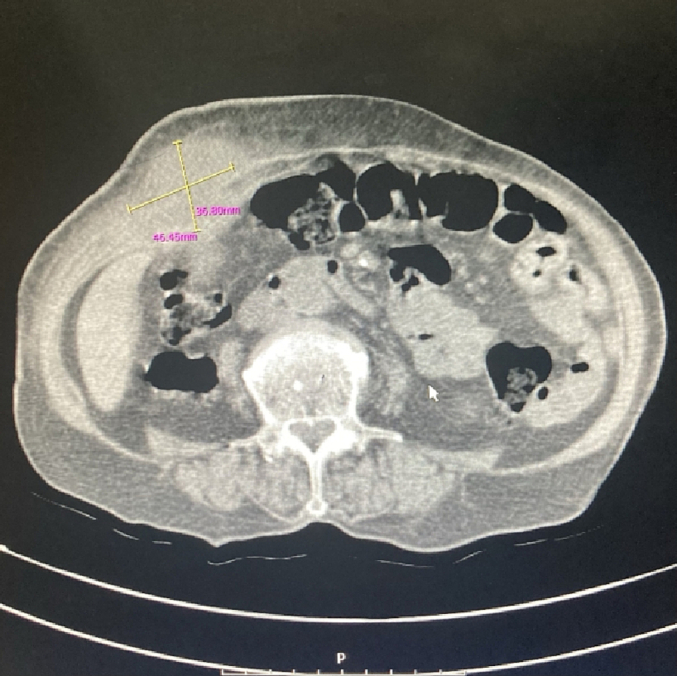
The bulged area at abdominal wall.

**Fig. 5:**
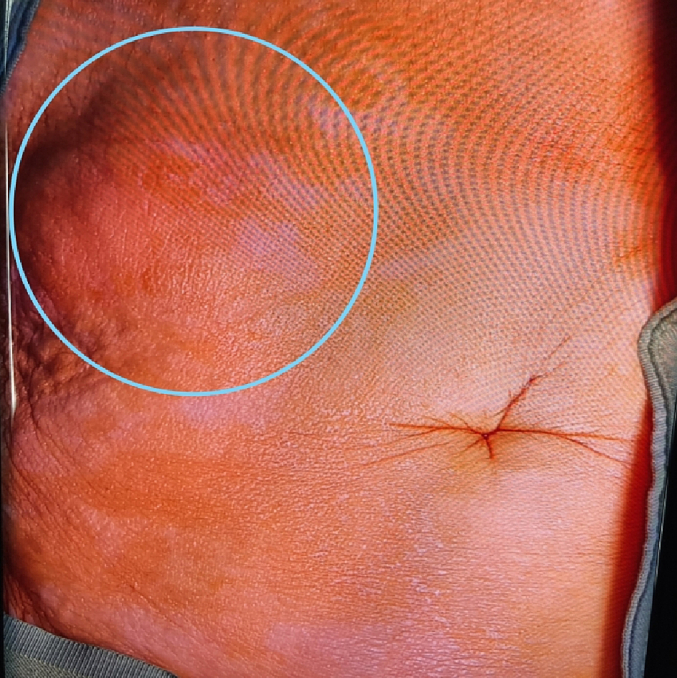
The bulged site at RUQ.

In the examination, a bulged and warm area was seen at the right upper abdomen with tenderness and positive rebound tenderness [Fig. 5]. DRE was the fecal stain. The bowel habit history was normal. Her vital signs were normal, but she was febrile. Lab tests showed mild leukocytosis, rises in creatinine and metabolic acidosis. Her lab results were WBC: 11.6/Hb: 12/Plt: 214/Neutr: 85/ESR: 40/CRP: 31/BS: 240/LFT: Nr/Amylase & Lipase: Nr/Bill Total & Direct: 1.2 & 0.4/LDH: 349/Urea: 59/Cr: 2.5. US sonography of abdomen showed a hypo-echo area on bulged site about 41 ∗ 43 mm and 15 cm^3^ surrounded by fat stranding with a neck in 5.5 mm diameter and mild collection. Prob-tenderness was positive, and the radiologist suggested a strangulated hernia. The next plan was a CT scan with IV contrast, but according to the patient's creatinine and probable AKI, a CT scan without contrast was taken and showed a 46 ∗ 36 mm hypodense instruction in RUQ which disseminated to the intraperitoneal area, with a mild rim of fluid around fondus of gallbladder and a 33 mm stone in fondus [Fig. 1, Fig. 2, Fig. 3, Fig. 4]. The examination and paraclinical results suggested gallbladder hernia and local perforation. So an urgent cholecystectomy was performed.

**Fig. 6:**
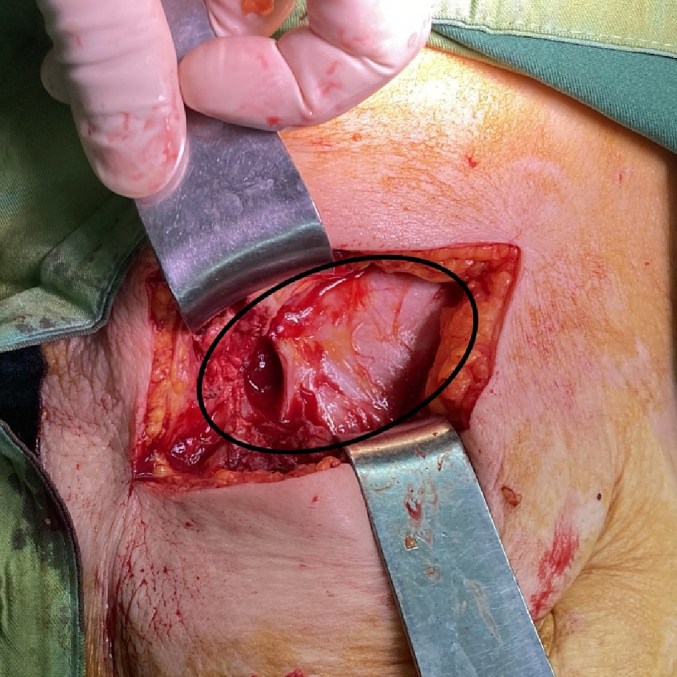
The perforated gallbladder in abdominal wall.

**Fig. 7:**
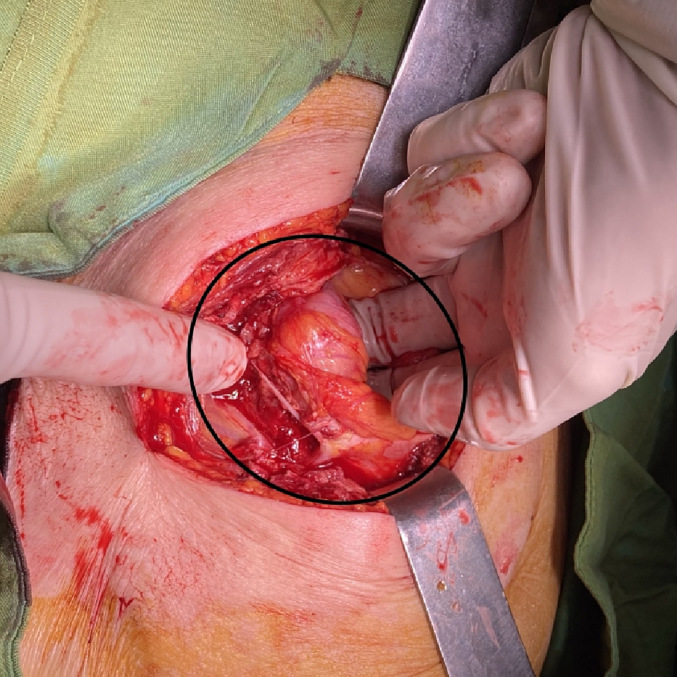
The gallbladder was released.

**Fig. 8:**
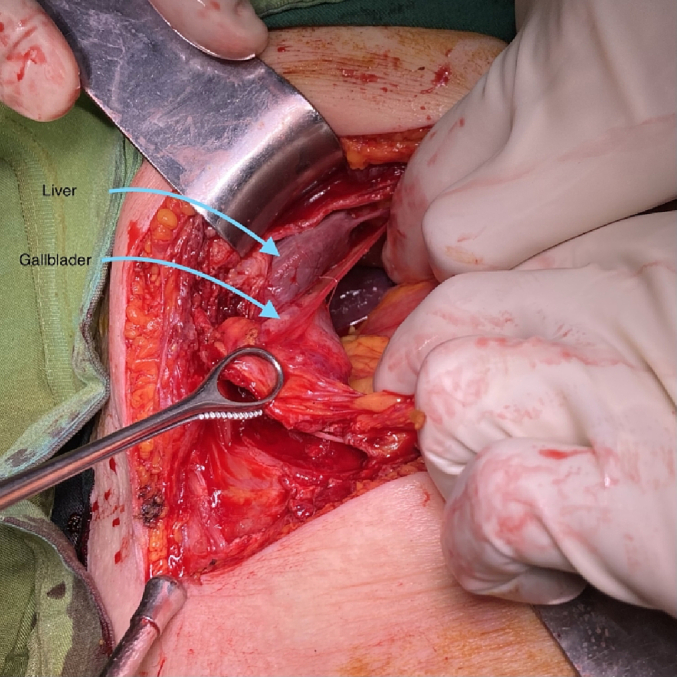
Cholecystectomy.

**Fig. 9:**
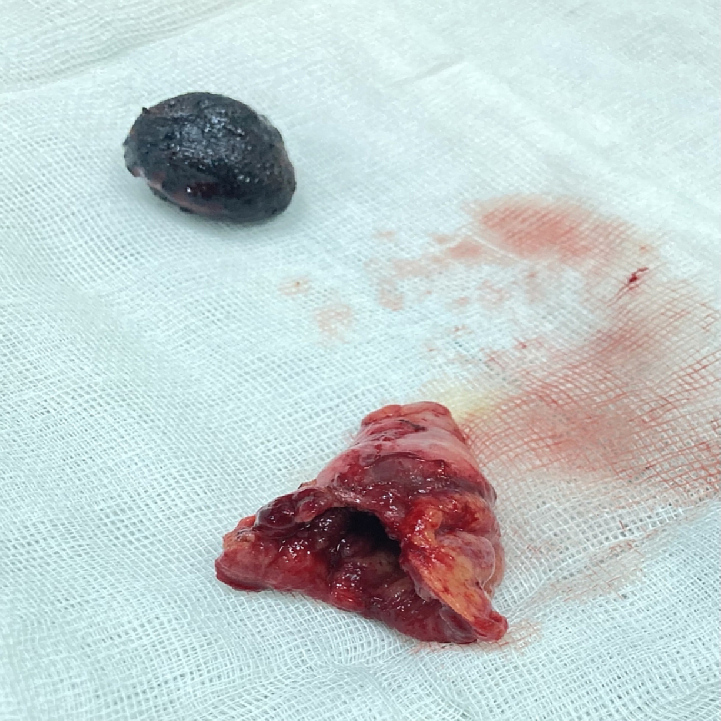
The gallbladder and gallstone.

### 2.3 Operation summary

The open cholecystectomy was performed under general anesthesia through the right kokher incision by an expert attending general surgeon and senior resident. In exploration, gallbladder was found herniated to the subcutaneous layer and perforated there with a few collections. The gallbladder was released from subcutaneous adhesions, and cholecystectomy was performed through the retrograde plan [Fig. 6, Fig. 7, Fig. 8]. The defect of herniation in the facia was irrigated and repaired. The operation was done without any complications. Postoperatively, medical treatment was followed by broad-spectrum intravenous antibiotics.

### 2.4 Outcome

Pathology reported chronic cholecystitis and lithiasis [Fig. 9]. There were no postoperative complications. The recovery was satisfactory and intestinal function was normal, she tolerated regular diet without nausea or vomiting, and leukocytosis decreased. The patient was discharged on the third postoperative day in good general condition.

## 3 Discussion

Gallbladder hernia is a rare event that happens more in elderly patients [1]. Based on our search in literature, it has been reported frequently as diaphragmatic herniations and intra-abdominal herniations like protrusion in the foramen of Winslow, but anterior abdominal wall or even inguinal and femoral hernia are still rare. Overall, it occurs more in elderly female patients.

In review of literature, we searched Pubmed, Scopus and Web of science for “Gallbladder AND Hernia” and “Gallbladder AND Herniation” from 1990 to 2023 in adults then we could find 65 related case reports. We selected the cases of gallbladder herniation through the abdominal wall and removed other hernia sites as diaphragmatic or internal herniations. In addition, we put the non-English language papers aside. The cases were divided into incisional and spontaneous. The majority of literature reported an incisional hernia. Ventral incisional hernia is a common complication of abdominal surgery with up to 20 % incidence, but incarceration of gallbladder is rare [9]. Most of the incisional herniations of gallbladder were parastomal (22 cases). One case was described as the evisceration of gallbladder at the site of a Pezzer drain [3], and six reports from various surgical sites. Spontaneous gallbladder herniation through the abdominal wall without any past acquired defect is extremely rare in adults, just reported six times before that [2,[4], [5], [6], [7], [8]].

The most related known causes of spontaneous gallbladder herniation are carcinoma, biliary tracked occlusion or abdominal wall weakness in elderly patients, respectively [2]. We have reported a spontaneous gallbladder hernia perforated in the subcutaneous layer without malignancy or biliary- tracked causes. The etiology of this condition is unclear, but there is some explanation for abdominal wall weakness due to probable prior cholecystitis and local inflammation in this case. *Pijpers* et al. raised that infiltration of the gall bladder can create a site of decreased local resistance in the abdominal wall as the progression of inflammatory reaction, abdominal wall muscles were involved, and a site of decreased local resistance was created in which the gall bladder mucosa herniated through the infiltrated abdominal wall [5].

The common presentations are abdominal pain and a firm irreducible mass or tender lump in the right upper abdomen with overlying erythema. Also, in case of perforated gallbladder, bilious staining may be seen [10]. The notable point is that cholecystitis presentation can be different in elderly or diabetic patients as asymptomatic, and the signs of inflammation as leukocytosis, fever or any reactions may happen later or not at all, so these patients need further evaluations.

However, imaging plays the main role in diagnosis and planning for probable operation. US sonography is the first and most feasible way for preliminary evaluations, but CT scan with IV and oral contrast is the choice to diagnose ventral herniations and provides additional information on the abdominal wall defect. Gallbladder herniation findings include the absence of the gallbladder from its anatomical position and a defined, thickened wall lesion within the hernia that does not enhance with oral contrast [1,2]. In addition, *Pijpers* et al. suggested that the MRI can be helpful in differentiation of muscular layers of the abdominal wall and the herniated gall bladder, exclude malignant characteristics and provide better results in an optimal surgical approach [5].

The first important thing in management due to experiences is to avoid reducing the ventral hernia if gallbladder herniation is suspected as there is a possibility of rupture and peritonitis [1]. According to our review, the majority of literature have recommended the laparoscopic approach for cholecystectomy and herniation repair in non-complicated ventral gallbladder hernia, but in case of cholecystitis or perforated gallbladder, open cholecystectomy is the only choice. In comparing the laparotomy and laparoscopy approach, the older reports have indicated more that the laparotomy via midline incision is preferred as it can bring better exposure and provide more accessibility for optimal operation. However, recent articles have noticed that laparoscopy is ideal for gallbladder hernia, especially in elderly patients as it can reduce postoperative morbidity and faster recuperation [2,5]. However, in this case, we preferred a classic kokher incision at the site of herniation due to subcutaneous perforation for irrigation and excision of necrotic tissues and repairing the defect by direct facial repair right through the incision. In reduction of an incarcerated gallbladder and cholecystectomy, decompression with a bore needle or aspiration and drainage of intra-luminal biliary sludge is also suggested at first as it can reduce the risk of contamination [1]. Some articles indicated that this can be managed conservatively, but we concern the patient would be at risk for rupture and resultant worsening sepsis if managed non-operatively, and we insist on operative management as soon as possible [9].

Another aspect is the repair of herniation, which is controversial to primary repair or follow up for later secondary surgery. We believe that the ventral gallbladder hernia must be repaired at the same time when the cholecystectomy is performed. The principle is reducing the content and repairing the abdominal wall defect by performing a mesh or structural repair. Performing a mesh is the first choice in non-complicated cases without contamination. However, acute cholecystitis or a ruptured gallbladder is a contraindication for mesh repair due to risk of infection from implanting a foreign body in a contaminated site [2]. However, *Trotta* et al. mentioned that using biological mesh is safe even in the presence of local sepsis, but it is not suitable for large defects as the results are as same as structural repair at a large defect [6,9]. Performing mesh reduces the chance of recurrence significantly, but it has some limitations as were said before. In a complicated case like this patient, direct fascial repair is a safe, simple, and rapid choice for emergency surgery and reducing the operation time, but it has associated high recurrence rates [10]. The parastomal herniation of the gallbladder is the most frequent and has some differences in management. According to the majority of literature, the wall defect should not be repaired because it can compromise the blood supply to the stoma. Some articles suggest the relocation of the stoma showing lower recurrences, but this prolongs surgery time and adds other complications [1,10].

## 4 Conclusion

Spontaneous ventral herniation of the gallbladder is an exceedingly rare condition. The use of imaging modalities is crucial in facilitating accurate diagnosis. Among these, CT scan with intravenous and oral contrast is considered the best option. It is important to note that reduction of the hernia should be avoided if gallbladder herniation is suspected. For cases that are not complicated, laparoscopic cholecystectomy and herniation repair are the preferred choices for management. However, for complicated, perforated or latent hernias, an open surgical approach is recommended, with flexibility in the choice of incision based on the herniation site and the surgeon's preference. Our recommendation is for cholecystectomy and hernia repair to be performed simultaneously and expeditiously in all cases, with conservative management not recommended at all.

## Consent

Written informed consent was obtained from the patient for publication of this case report and accompanying images. A copy of the written consent is available for review by the Editor-in-Chief of this journal on request.

## Ethical approval

Ethical approval is exempt/waived at our institution.

Shahid Beheshti University of Medical Sciences.

## Funding

This article did not receive fund.

## Author contribution

Dr. Majid Samsami is the main author and he has designed this report. The Associate Professor.

Dr. Seyed Pedram Kouchak Hosseini participated in Conceptualization.

Dr. Hojatolah khoshnoudi participated in Methodology.

Dr. Mohammad Aghaei participated in Investigation and Term.

Dr. Fatemeh Parsaeian participated in Data curation.

Dr. Alireza Haghbin Toutounchi is the writer of this article and corresponding author.

## Guarantor

Dr. Majid Samsami accepts all responsibility of this article.

## Research registration number

Not applicable.

## Declaration of competing interest

All authors declare that they have no conflicts of interest.
